# Breast cancer treatment in clinical practice compared to best evidence and practice guidelines

**DOI:** 10.1038/sj.bjc.6601439

**Published:** 2004-01-06

**Authors:** B S Bloom, N de Pouvourville, S Chhatre, R Jayadevappa, D Weinberg

**Affiliations:** 1 Division of Geriatrics, Department of Medicine, University of Pennsylvania; 2 Leonard Davis Institute of Health Economics, Philadelphia, PA, USA; 3 Université de Paris V, Hôpital Cochin, Paris, France; 4 Fox Chase Cancer Center, Philadelphia, PA, USA

**Keywords:** breast cancer treatment, evidence-based medicine

## Abstract

There is sparse evidence on community practice patterns in treating women with breast cancer. This study compared care of women with breast cancer with evidence from meta-analyses and US National Comprehensive Cancer Network (NCCN) clinical guidelines. Records of 4395 women with breast cancer were abstracted from practices of 19 surgeon oncologists in six specialty practices in the Philadelphia region during 1995–1999. Patients were followed through December 2001. Low-frequency data were obtained on all patients. All other data were from a random sample of 464 women, minimum of 50 patients per practice. Actual care provided was compared to NCCN guidelines and results of meta-analyses. Fewer than half the women received treatments reflecting meta-analysis results or NCCN guidelines, by disease stage/TNM status. Adherence to either standard varied from 0% for LCIS to 87% for stages IIA or IIB node positive. There are multiple interactive reasons for low adherence to guidelines or meta-analyses results, including insufficient health system supports to clinicians, inadequate organisation and delivery systems and ineffective continuing medical education. The paucity of written information from patient records on physician/patient interactions limits the understanding of treatment decisions.

Breast cancer outcomes have improved over time because of more effective treatments, greater public awareness and earlier diagnosis (Smart *et al*, 1997). Factors contributing to outcomes variability include unequal access to care (Mandelblatt *et al*, 1999), age (Bailar and Gornik, 1997), ethnicity and income (Roetzheim *et al*, 2000), patient treatment preferences (Ashcroft *et al*, 1985; Contant *et al*, 2000; Mandelblatt *et al*, 2000), surgeon preferences (Mor *et al*, 2000) and geography (Farrow *et al*, 1996; Coleman *et al*, 1999; Polednak, 2000; Morrow *et al*, 2001). The objective of this study was to compare actual care with published guidelines and best evidence from meta-analyses.

## METHODS AND MATERIALS

### Study site and patient accrual

Six single-specialty surgical oncology practices in northeastern US with 19 oncology surgeons participated. Patients entered the study after diagnosis of breast cancer and operation between 1 January 1995 and 31 December 1999, and were followed through 31 December 2001. None were in clinical trials.

We used two sampling methods to collect data. From the total patient population (*n*=4395) we collected specific low-frequency data determined by study pretest. Next, we selected a random sample (*n*=464) from each practice based on volume of breast cancer patients, with a minimum of 50 patients from each practice.

Utilisation of five treatments were selected because each were included in clinical practice guidelines and confirmed by meta-analyses:

1. breast-conserving surgery (BCS) and mastectomy;
2. breast reconstruction following operation;
3. adjuvant cytotoxic chemotherapy;
4. radiation;
5. nonsteroidal antioestrogens.

### Treatment algorithms

We chose four treatment algorithms from two sources. Treatment recommendations by both were the same or similar by diagnosis and stage. The first comparator was the US National Comprehensive Cancer Network (NCCN) clinical practice guidelines (NCNN, 1996). The second was derived from treatment-specific meta-analyses of optimal care for each clinical scenario by diagnosis/TNM/stage. We used only meta-analyses published before 1996 as 1995 was the beginning of patient enrollment.

#### Breast-conserving surgery or mastectomy

##### NCCN guideline

For DCIS, LCIS and stages I and node-negative IIa, BCS with negative margins, BCS plus radiation, or mastectomy without lymph node dissection, are equally appropriate, unless the woman chooses otherwise.

##### Meta-analyses: 

Bradley *et al* (1990)found similar mortality for women treated with mastectomy or BCS for early stage DCIS and LCIS despite higher recurrence rates with BCS.

#### Breast reconstruction

##### NCCN guideline

All women should have the option of breast reconstruction following mastectomy.

##### Meta-analyses: 

There were no meta-analyses and limited results from controlled research (Harcourt and Rumsey, 2001). NCCN recommends BCS, and patient preference (Ashcroft *et al*, 1985; Mandelblatt *et al*, 2000), satisfaction (Contant *et al*, 2000), psychological impact (Noon *et al*, 1982; Schain *et al*, 1984; Stevens *et al*, 1984) and self-image (Sneeuw *et al*, 1992; Al-Ghazal *et al*, 2000) show its importance to women.

#### Radiation therapy

##### NCCN guideline

All women with DCIS and negative margins, and all women with BCS or mastectomy with T3 and greater should have radiation of standard fractionated doses totaling 40–60 Gy, unless contraindicated and/or the woman refuses.

##### Meta-analyses: 

The Early Breast Cancer Trialists' Collaborative Group (EBCTCG) found reduced relapse rates and comparable mortality for radiation following operation regardless of stage and node status EBCTCG, 1995). Radiation and BCS for DCIS reduces recurrence by 50% (Bradley *et al*, 1990; Boyages *et al*, 1999).

#### Cytotoxic chemotherapy

##### NCCN guideline

Multicycle adjuvant cytotoxic polychemotherapy should be prescribed for all women with node positive disease and/or with tumour greater than 1 cm, except for DCIS and LCIS, unless the woman refuses.

##### Meta-analyses: 

Himel *et al* (1986) found significant survival benefits with cytotoxic polychemotherapy. EBCTCG (1988, 1992) concluded that it reduced annual risk of death by 16% and disease recurrence by 28%, irrespective of stage and node status .

#### Nonsteroidal antioestrogens

##### NCCN guideline

Women with positive oestrogen receptor (OR) and/or progesterone receptor (PR) disease, irrespective of diagnosis and disease stage, should receive a nonsteroidal antioestrogen unless contraindicated and/or the woman refuses.

##### Meta-analyses: 

EBCTCG (1988, 1992)found that tamoxifen reduced mortality and disease recurrence for women with OR/PR-positive disease. Combining chemotherapy with tamoxifen reduced 10-year relative mortality risk by 30–40%.

#### Other interventions

Axillary node dissection was included in treatment algorithms because NCCN recommended it in their guidelines even though no meta-analysis was published until 1999 (Orr, 1999). Modified radical mastectomy or lumpectomy plus axillary node dissection were also included.

#### Combined treatment algorithms

Four scenarios were developed from NCCN guidelines and meta-analyses. Each algorithm from both sources was compared to actual patient treatment. It is to be noted that a treatment prescribed by the physician that followed either algorithm was considered appropriate even if the women refused or did not complete therapy.

## RESULTS

### Patient characteristics

There were 4395 women in the entire study and 464 women (10. 6%) in the study random sample. Patient age was uniformly distributed from ⩽45 to >80 years (Table 1

**Table 1:** Patient age and ethnicity at breast cancer diagnosis

**Age**	**Total**	**% White**	**% Non-White**	**% Unknown**
<45	13.6	58.7	19.1	22.2
45–49	11.0	60.8	15.7	23.5
50–54	10.8	58.0	14.0	28.0
55–59	10.1	76.6	14.9	8.5
60–64	8.2	60.5	10.5	29.0
65–69	11.6	57.4	20.4	22.2
70–74	9.7	75.6	8.9	15.6
75–79	9.3	65.1	20.9	14.0
80–84	5.2	79.2	8.3	12.5
85–89	1.7	62.5	0	37.5
90+	0.6	100.0	0	0
Unknown	8.2	63.2	15.8	21.0
Total %	100.0	64.7	15.0	20.3
				
Mean: 60.25	s.d. 13.77			
Skewness: 0.077				
Min: 24	Max: 94			

). Ethnicity mirrored the region's population except for under representation of Hispanics. Every woman in the study had health insurance, and 51.9% had known ambulatory pharmaceutical coverage.

### Physician characteristics

All 19 study physicians were board certified general surgeons and specialized in breast cancer care. In all, two-thirds were male and 17 were Caucasian; all practices were partnerships.

Nearly all patients had numerous consultations with multiple physicians and others in addition to their surgeon, for example, radiation oncologists, psychiatrists and nutritionists. About 85% continued regular visits to the surgeon from study entry to study completion or death.

### Disease characteristics

#### Diagnostic testing

Use of diagnostic tests recommended by NCCN varied – OR/PR status (81.6% of women), HER/2 neu oncogene (2.2%), Ki67 (27.6%) and BCRA1/BCRA2 (0.2%). Except for use of OR/PR status, none were included in treatment algorithms.

Based on TNM status, most women (82.4%) had early stage disease (0, I or IIa) (Table 2

**Table 2:** Disease stage at diagnosis (*N*=464)

**Stage**	**Percent**
Stage 0,I	55.0
Stage IIa	27.4
Stage IIb	8.6
Stage IIIa	3.7
Stage IIIb	3.0
Stage IV	2.4
	
Total	100.1

), similar to US national data (Lazovich *et al*, 1991).

### Utilisation of five individual treatments

#### Breast-conserving surgery

Breast-conserving surgery was provided to 47.1% of women in the study sample (*n*=464) (Table 3

**Table 3:** Percent of women prescribed individual optimal or NCCN recommended treatments, by diagnosis and disease stage (*N*=464)

		**Diagnosis and stage**		
**Treatment**	**DCIS, stage I or IIa node neg (*n*=354)**	**LCIS (*n*=14)**	**IIa or IIb node pos (*n*=34)**	**IIIA, IIIB, IV (*n*=61)**
Lumpectomy	64.2	33.3	17.4	14.3
Axillary node dissection	N/A	N/A	8.7	7.1
Sentinal node biopsy	N/A	N/A	8.7	2.4
Simple/modified radical mastectomy	10.9	N/A	73.9	67.4
Reconstruction^a^	4.2	33.3	13.0	19.0
Radiation	60.4	30.4	39.1	40.5
Chemotherapy	19.2	N/A	34.8	26.2
Tamoxifen, if OR and/or PR positive	79.5	66.7	56.5	60.6

a Following any mastectomy. N/A=not applicable.

), higher than results from a national US study (42.6%) (Morrow *et al*, 2001). Fewer than 1% refused lumpectomy and chose mastectomy. The majority (69.5%) of women with node-positive disease had modified radical mastectomy regardless of disease stage. Among the 85 women with disease recurrence (*n*=4395), 32.9% had lumpectomy and the remainder mastectomy.

#### Breast reconstruction

Breast reconstruction was provided to 20.8% of women within 1 year after mastectomy (Table 3), nearly two-fold higher than reports of the same period (Polednak, 2000; Morrow *et al*, 2001). In addition, 1.5% of women with lumpectomy had breast reconstruction.

#### Radiation therapy

Radiation was provided to 56.8% of women (Table 3). Over 90% received recommended fractionated doses totaling 40–60 Gy (28); 98% completed their regimen. Fewer than 1% refused radiation.

#### Cytotoxic polychemotherapy

Cytotoxic polychemotherapy was given to 37.1% of eligible women (Table 3). A total of 19 (4.1%) had neoadjuvant plus adjuvant regimens. Two refused all chemotherapy.

#### Nonsteroidal antioestrogens

Of women with OR- and/or PR-positive disease, 80.4% received a nonsteroidal antioestrogen, nearly always tamoxifen, including the 8% who refused (Table 3). Most who refused did so because of potential side effects. Nearly 5% were diagnosed with recurrent breast cancer while on tamoxifen for secondary prophylaxis.

Among women with recorded previous history of deep venous thrombosis (DVT) or pulmonary embolus (PE) (*n*=95 of the total population of 4395), 60.0% (*n*=57) got tamoxifen, of whom 83.3% (*n*=47) experienced another DVT or PE. All were OR or PR positive; none died. None were on recorded anticoagulation therapy.

#### Physician adherence with optimal or NCCN guideline treatments

Optimal or NCCN treatments were provided to 45.0% of women (Table 4

**Table 4:** Prescribing optimal or NCCN clinical guideline treatment (*N*=464)

**Clinical scenario**	
(*1) DCIS, and/or stage I, or IIA node negative (N=354)*	
*Meta analysis optimal treatment*: lumpectomy, radiation and tamoxifen if OR and/or PR positive, unless the woman refuses	44.7%
*NCCN practice guidelines*: mastectomy with unclear disease margins, or BCS with negative margins plus radiation, plus breast reconstruction and tamoxifen if OR positive, unless the woman refuses	59.6%
(*2) LCIS (N=14)*	
*Meta analysis optimal treatment*: lumpectomy, radiation and tamoxifen if OR and/or PR positive, unless the woman refuses	14.2%
*NCCN practice guidelines*: observation alone, or bilateral mastectomy plus reconstruction, unless the woman refuses	0%
(*3) Stage IIa or IIb node positive (N=34)*	
*Meta analaysis optimal treatment and NCCN practice guidelines*: BCS with/without axillary node dissection or sentinal node biopsy, or modified radical mastectomy plus reconstruction, radiation, polychemotherapy, and tamoxifen if OR and/or PR positive, unless the woman refuses	14.7%
(*4) Stage IIIa, IIIB, or IV (N=61)*	
*Meta analysis optimal treatment*: BCS with/without axillary node dissection, or modified radical mastectomy plus breast reconstruction, and radiation, polychemotherapy and tamoxifen if OR and/or PR positive, unless the woman refuses	42.2%
*NCCN practice guidelines*: modified radical mastectomy plus breast reconstruction, radiation, polychemotherapy, and tamoxifen if OR and/or PR positive, unless the woman refuses	11.8%

). The majority at every disease stage, except those with DCIS, was not treated according to NCCN practice guidelines or optimal regimens from meta-analyses. Overuse of mastectomy, and under use of lumpectomy, reconstruction, radiation and cytotoxic polychemotherapy were the main results for every disease stage and scenario.

### Factors associated with service use

We tested by logistic regression all independent patient, physician, disease and treatment variables to determine which influenced treatment variations. Patient age was inversely related with breast reconstruction (*P*=0.006), cytotoxic polychemotherapy (*P*<0.001) and radiation (*P*=0.02). Patient age (*P*<0.0001) was directly, and surgeon female gender (*P*=0.007) inversely, related to tamoxifen use.

Odds ratios (ORs) analyses found that patient age was inversely related to radiation (OR=0.98, CI .963–.997) and cytotoxic polychemotherapy (OR=0.91, CI 0.891–0.938), and directly related to tamoxifen use (OR=1.04, CI 1.02–1.05). Surgeon female gender was inversely related to tamoxifen (OR=0.51, CI=0.312–0.837). No differences from either analysis were clinically significant.

Cluster analysis of individual treatments by physician, using fixed-effects and random-effects models, found no significant associations with independent variables. Thus, no variables had important systematic effect on treatment variation from NCCN guidelines and meta-analyses.

## DISCUSSION

Most women with breast cancer in this study were not treated according to regimens from either meta-analyses or NCCN clinical practice guidelines. In only one scenario (DCIS) was the majority of women treated according to either standard. These findings are consistent with a 1998 study of physician adherence with US National Institutes of Health clinical practice guidelines for early stage breast cancer, and with a 2002 study of all beneficial breast cancer diagnostic and therapeutic interventions (Lazovich *et al*, 1991; Malin *et al*, 2002).

Patient and physician characteristics had inconsistent relation to treatment. Physician-specific treatment at the beginning was essentially the same as at the end of the study, suggesting that incorporation of best evidence and clinical practice guidelines is slow in altering clinical care patterns. However, available meta-analyses published by 1995 may not have been available long enough to influence change.

Over- and undertreatment occurred simultaneously, sometimes in the same patient. For example, excluding women who requested it, 15.1% with DCIS or stage I, with clear margins, had mastectomy, an example of over treatment based on NCCN guidelines and meta-analyses; and only 15.6% had breast reconstruction within a year, an example of under treatment. In another example, tamoxifen was provided to 60% of women with known history of DVT or PE, a contraindication for use. Lack of specifics on detailed discussions between patient and physician means we do not know how and why decisions on specific care modalities were made.

What might account for discordance between actual and recommended or optimal treatment? First, there are likely important differences between patients in randomized control trials and general patients treated in the community. Community patients have variable disease and personal preferences, and physicians tailor treatment to individual needs. An unanswered question is whether physicians who participate in randomised trials practice according to their own study results, and differently than physicians not participating in trials.

Second, two recent reports by the US Institute of Medicine (IOM) clearly noted health system organisation, delivery and financing deficiencies, results likely applicable to many countries (Institute of Medicine, 2001, 2002). There are inappropriate incentives and inadequate supports and mechanisms to help physicians and patients understand, choose and adhere to treatments with the greatest likelihood of benefit. Tying patient care processes to predefined outcomes may provide impetus for change (Institute of Medicine, 2001). Any recommendations for changes in physician practice must be coupled with increased patient participation and adherence to prescribed care (Morrison *et al*, 2000).

An equally knotty problem is physician continuing medical education (CME) to incorporate best scientific evidence into clinical practice. Commonly used CME methods like lectures and distributing printed materials alone have little or no success in changing physician clinical practice. Other techniques, like group interactive learning and reminders and audit and feedback are highly effective, but infrequently used (Grimshaw *et al*, 1994; Thomson O'Brien *et al*, 2002).

### Study limitations

The most serious study limitation was lack of written information on patient and physician discussions and treatment choices. Next, a patient cohort treated in one geographic region may not be representative of all women with breast cancer and thus may reduce external validity.
